# A Novel Nomogram Based on a Competing Risk Model Predicting Cardiovascular Death Risk in Patients With Chronic Kidney Disease

**DOI:** 10.3389/fcvm.2022.827988

**Published:** 2022-04-12

**Authors:** Ning Li, Jingjing Zhang, Yumeng Xu, Manshu Yu, Guowei Zhou, Yawei Zheng, Enchao Zhou, Weiming He, Wei Sun, Lingdong Xu, Lu Zhang

**Affiliations:** Affiliated Hospital of Nanjing University of Chinese Medicine, Jiangsu Province Hospital of Chinese Medicine, Nanjing, China

**Keywords:** nomogram, chronic kidney disease, competing risk model, cardiovascular death, prediction model

## Abstract

**Objective:**

Chronic kidney disease (CKD) patients are more likely to die from cardiovascular disease (CVD) than develop renal failure. This study aimed to develop a new nomogram for predicting the risk of cardiovascular death in CKD patients.

**Methods:**

This study enrolled 1656 CKD patients from NHANES 2003 to 2006 survey. Data sets from 2005 to 2006 survey population were used to build a nomogram for predicting the risk of cardiovascular death, and the nomogram was validated using data from 2003 to 2004 survey population. To identify the main determinants of cardiovascular death, we performed univariate analysis and backward-stepwise regression to select the key factors. The probability of cardiovascular death for each patient in 5, 7, and 9 years was calculated using a nomogram based on the predictors. To assess the nomogram’s performance, the area under receiver operating characteristic curve (AUC) and the calibration curve with 1,000 bootstraps resamples were utilized. The prediction model’s discrimination was examined using cumulative incidence function (CIF).

**Results:**

Age, homocysteine, potassium levels, CKD stage, and anemia were included in the nomogram after screening risk factors using univariate analysis and backward-stepwise regression. Internal validation revealed that this nomogram possesses high discrimination and calibration (AUC values of 5–, 7–, and 9-years were 0.79, 0.81, and 0.81, respectively). External validation confirmed the same findings (AUC values of 5–, 7– and 9-years were 0.76, 0.73, and 0.73, respectively). According to CIF, the established nomogram effectively differentiates patients at a high risk of cardiovascular death from those at low risk.

**Conclusion:**

This work develops a novel nomogram that integrates age, homocysteine, potassium levels, CKD stage, and anemia and can be used to more easily predict cardiovascular death in CKD patients, highlighting its potential value in clinical application.

## Introduction

Chronic kidney disease (CKD) has developed into a global health crisis, lowering patients’ quality of life declining and resulting in disability (1). At present, there are a large number of CKD patients worldwide. According to statistics, 42 million of Americans suffer from CKD (2), increasing to 700 million people globally (3). CKD has a slowly progressive clinical course and results in irreversible deterioration in renal function. Along with kidney function deterioration, the risk of cardiovascular (CV) complications also increases. According to the 2016 ESC/EAS guidelines (4), patients with stages 3–5 CKD are at high risk of experiencing CV adverse events. Additionally, the 2016 USRDS annual data report (2) indicates that CKD patients are more likely to experience CV events than undergoing dialysis. Cardiovascular disease (CVD) is also a leading cause of death in CKD patients. About 45.1% of people who reach stage 3 CKD will die of CV causes, and the mortality rate increases to 58% at stage 5 (5, 6). Given the high incidence and mortality of CV events, global attention has shifted to improving the prediction of cardiovascular outcomes in CKD patients.

Although adverse cardiovascular outcomes are common in CKD patients, there is currently no reliable tool to predict them (7). Constructing an accurate prediction model for cardiovascular outcomes is critical for clinical decision-making. It can not only identify high-risk groups and improve their prognosis but also reduce excessive medical expenses for low-risk patients. Currently, nomograms have emerged as a promising method for predicting adverse outcomes in kidney diseases. It takes into account a variety of factors, including demographics, biomarkers, health status, and behaviors, and can classify patients into different clinically distinguishable clusters linked to different outcomes, such as CKD progression, CVD, and death. It is a good way to group patients into different subtypes based on their baseline features, which is more helpful for the realization of precision medicine and individualized medicine. Therefore, this study aims to establish a visualized prediction model, scoring cardiovascular death risk in CKD patients using a nomogram.

## Materials and Methods

### Patients and Predictors

In this study, the data of enrolled patients were obtained from the 2003 to 2006 National Health and Nutrition Examination Survey (NHANES). The population from 2005 to 2006 was used to construct the model, whereas the population in 2003–2004 served as an external validation cohort. The inclusion criteria include the following: (1) patients with CKD [defined as estimated glomerular filtration rate (eGFR; eGFR calculation was based on CKD-EPI formula) <60 mL/min per 1.73 m^2^ or urinary albumin-to-creatinine ratio (UACR) >30 mg/g]; (2) patients with 18–75 years old. Patients with incomplete clinical data or lost contact during the follow-up period were excluded. Based on our clinical experience and literature review, we selected some risk factors that might affect cardiovascular prognosis, including diabetes, hypertension, gender, age, glycosylated hemoglobin (HbA1c), blood urea nitrogen (BUN), serum phosphorus (P), serum potassium, serum uric acid (UA), UACR, alanine aminotransferase (ALT), aspartate aminotransferase (AST), serum total cholesterol (TC), serum total triglycerides (TG), calcium (Ca), Body Mass index (BMI), albumin (Alb), C-reactive protein (CRP), homocysteine, CKD stage (eGFR < 60 mL/min per 1.73 m^2^ or eGFR ≥ 60 mL/min per 1.73 m^2^), and anemia (defined as hemoglobin <13 g/dL for men and <12 g/dL for women). The information of questionnaire was used to determine the presence of complications.

### Prespecified Outcomes

Cardiovascular death was the main prespecified outcome. Since the occurrence of cardiovascular events in CKD patients may be a long-term process, we measured the probability of cardiovascular deaths at three different time points (5–, 7–, and 9-year). The situation of cardiovascular death and the follow-up time were extracted from the public-use linked mortality file from the National Center for Health Statistics (NCHS) and matched with patients’ ID from NHANES database. The follow-up time is defined as the interval between the interview date and the death date.

### Statistical Analysis

The three-knot cubic spline (10, 50, and 90%) was employed to account for potential non-linear effects of continuous data. Shapiro-Wilk method was utilized to examine the normal distribution of continuous data. The continuous variables conformed to normal distribution were compared using independent samples *t*-test and are presented as mean ± standard deviation (SD). Mann-Whitney *U*-test was used to determine the non-normally distributed variables, and they were presented as median (1st–3rd quartile). Chi-square tests were performed to compare categorical variables. If the theoretical frequency was less than 10, Fisher’s exact test was preferred. Given the possibility of competitive risk, relying solely on one endpoint-analysis approach can lead to an inaccurate assessment of the probability of adverse events. Compared with Cox proportional hazards model, the competitive risk model can provide a more accurate estimate of the cumulative incidence of multiple outcomes (8). Therefore, in this study, we use a competing risk model for analysis, defining cardiovascular mortality as the primary outcome and other causes of death as competing events. First, fine and gray regression models were used for univariable analysis (9); variables with a statistical significance of the estimated regression coefficients of *P* ≥ 0.2 were removed. Second, significant variables were selected using backward-stepwise regression (10), and the model with the lowest akaike information criterion (AIC) value was used to construct the competing risk nomogram (11). To evaluate the relative risk of each model, sub-distribution hazard ratios (SHRs) and 95% confidence intervals (95% CIs) were determined. The receiver operating characteristic (ROC) curves (12) were deployed to evaluate the sensitivity and specialty of the established model. The model’s discrimination ability is determined using the area under the curve (AUC) at different time points. Larger AUC values indicate better overall discrimination. The nomogram’s accuracy was measured using a calibration curve with 1,000 bootstrap resamples (13). Decision curve analysis (DCA) was conducted to determine the nomogram’s clinical utility by quantifying the net benefits at different threshold probabilities (14). For external validation, we calculated the total point of each patient according to an established nomogram and used it as a factor in the competing risk model. AUC and calibration curve were performed to verify the model’s external applicability. To better assess the model’s discrimination ability, we calculated the total score for each patient and divided them into high- and low-risk groups based on their median score. Then, CIF curves (cumulative incidence function) were constructed using Gray’s test (15) to explore the risk differences between high- and low-risk groups. In all analyses, *P* < 0.05 (double) was considered statistically significant.

All statistical analyses were performed using R software version 4.05.^1^ R software was used to implement the three-knot cubic spline (survminer package), competing risk model (mstate, riskRegression package), nomogram (survival package), CIF (cmprsk Package), ROC curve (timeROC), AUC, and calibration plot.

## Results

### Baseline Characteristics

According to our prespecified inclusion and exclusion criteria, this study enrolled 1656 CKD patients. Among them, 10.5 and 15.4% died from CVD in training and validation cohorts, accounting for 33 and 35% of the total deaths, respectively. The median ages in training and validation cohorts were 66 and 70 years, respectively. The median follow-up times were 121 and 142 months in training and validation cohorts, respectively. In the training cohort, 53.3% of participants were males, 22.41% had diabetes, and 56.23% had hypertension. In the validation cohort, 53.71% were males, 25.42% had diabetes, and 59.18% had hypertension. Patients with stage 3–5 CKD account for 38.74% in training cohort and 37.82% in validation cohort. According to the observation of clinical characteristics, age, potassium, AST, homocysteine, and sodium levels were significantly different between the two cohorts (*P* < 0.05). Table 1 summarizes laboratory and clinical characteristics of patients.

**TABLE 1:** Baseline characteristics of the training cohort and validation cohort.

Variables	Training cohort (*n* = 955)	Validation cohort (*n* = 661)	*P*-value
Age (years)	66.00 [50.00–77.00]*^a^*	70.00 [54.00–80.00]	**0.002**
Gender (*n*, %)			0.872
Female	446 (46.70%)*^b^*	306 (46.29%)	
Male	509 (53.30%)	355 (53.71%)	
BMI (*n*, %)			0.68
<24 Kg/m^2^	243 (25.45%)	180 (27.23%)	
24–27 Kg/m^2^	199 (20.84%)	139 (21.03%)	
≥28 Kg/m^2^	513 (53.72%)	342 (51.74%)	
Hypertension (*n*, %)			0.239
Yes	537 (56.23%)	392 (59.18%)	
No	418 (43.77%)	269 (40.82%)	
Diabetes (*n*, %)			0.162
Yes	214 (22.41%)	168 (25.42%)	
No	741 (77.59%)	493 (74.58%)	
Anemia (*n*, %)			0.225
No	801 (83.87%)	569 (86.08%)	
Yes	154 (16.13%)	92 (13.92%)	
AST (U/L)	24.00 [20.00–28.00]	23.00 [20.00–27.00]	**0.028**
TC (mmol/L)	5.00 [4.00–6.00]	5.00 [4.00–6.00]	0.119
Homocysteine (*n*, %)			**<0.001**
≥15.3 umol/L	852 (89.21%)	529 (80.03%)	
<15.3 umol/L	103 [10.79%]	132 (19.97%)	
CRP(*n*, %)			0.389
<1.8 mg/dL	830 (86.91%)	584 (88.35%)	
≥1.8 mg/dL	125 (13.09%)	77 (11.65%)	
HbA1c (*n*, %)			0.447
<6.0%	649 (67.96%)	461 (69.74%)	
≥6.0%	306 (32.04%)	200 (30.26%)	
Albumin (*n*, %)			0.472
<40 g/L	308 (32.25%)	202 (30.56%)	
≥40 g/L	647 (67.75%)	459 (69.44%)	
ALT(*n*, %)			0.747
<35 U/L	849 (88.90%)	591 (89.41%)	
≥35 U/L	106 (11.10%)	70 (10.59%)	
BUN(*n*, %)			0.709
<8.6 mmol/L	824 (86.28%)	566 (85.63%)	
≥8.6 mmol/L	131 (13.72%)	95 (14.37%)	
Phosphorous(*n*, %)			0.269
<1.5 mmol/L	476 (49.84%)	311 (47.05%)	
≥1.5 mmol/L	479 (50.16%)	350 (52.95%)	
TG (*n*, %)			0.785
<1.3 mmol/L	362 (37.91%)	255 (38.58%)	
≥1.3 mmol/L	593 (62.09%)	406 (61.42%)	
Sodium (*n*, %)			**0.028**
<140 mmol/L	527 (55.18%)	328 (49.62%)	
≥140 mmol/L	428 (44.82%)	333 (50.38%)	
Potassium (*n*, %)			**<0.001**
<3.5 mmol/L	44 (4.61%)	42 (6.35%)	
3.5–5.0 mmol/L	777 (81.36%)	599 (90.62%)	
≥5.0 mmol/L	134 (14.03%)	20 (3.03%)	
UACR (*n*, %)			0.991
≤30 mg/g	448 (46.91%)	309 (46.75%)	
31–300 mg/g	425 (44.50%)	294 (44.48%)	
>300 mg/g	82 (8.59%)	58 (8.77%)	
CKD. Stage (*n*, %)			0.708
eGFR < 60 mL/min per 1.73 m^2^	370 (38.74%)	250 (37.82%)	
eGFR ≥ 60 mL/min per 1.73 m^2^	585 (61.26%)	411 (62.18%)	
CVD. Death (*n*, %)			**0.003**
No	855 (89.53%)	559 (84.57%)	
Yes	100 (10.47%)	102 (15.43%)	

*^a^Weighted median [95% CI for median].*

*^b^Actual frequency (weighted percentage).*

*eGFR, estimated glomerular filtration rate; BMI, body mass index; CRP, C-reactive protein, HbA1c: glycosylated hemoglobin; ALT, alanine aminotransferase; AST, aspartate aminotransferase; BUN, blood urea nitrogen; TG, serum total triglycerides; TC, serum total cholesterol; CVD, cardiovascular disease. Bold values indicate the results were statistical significance.*

### Cardiovascular Death Risk Prediction Models

Following univariate analyses of all variables using fine and gray regression models, ten significant variables in univariate analysis (*P* < 0.2) were entered into multivariate competing risk model (Table 2). Five variables (age: SHR 1.09, [95% CI: 1.06–1.11], homocysteine: SHR 1.83, [95% CI: 1.12–2.99], CKD stage: SHR 0.61, [95% CI: 0.39–0.94], anemia: SHR 1.67, [95% CI: 1.08–2.58], and potassium: middle/low: SHR 0.57, [95% CI: 0.28–1.14], high/low: SHR 0.40, [95% CI: 0.18–0.90]) were retained after backward-stepwise selection with the lowest AIC. We randomly selected a patient to calculate the probability of cardiovascular death. By summing up the scores of various risk factors, we can calculate that the cardiovascular death probabilities of patients in 5, 7,and 9 years are 3.63, 5.94, and 8.01% respectively.

**TABLE 2:** Univariate and multivariate fine and gray competing risk regression analyses.

Variables	Univariate analysis		Multivariate analysis (Stepwise model)	
	SHR (95% CI)	*P*-value	SHR (95% CI)	*P*-value
Age (years)	1.08 (1.06–1.10)	<0.01	1.09 (1.06–1.11)	**<0.01**
**Gender**				
Female	Ref	–		
Male	0.73 (0.49–1.08)	0.12		
**BMI**				
<24 Kg/m^2^	Ref	–		
≥24 Kg/m^2^	0.83 (0.66–1.04)	0.11		
**Hypertension**				
No	Ref	–		
Yes	0.65 (0.43–0.98)	0.04		
**Diabetes**				
No	Ref	–		
Yes	1.08 (0.67–1.75)	0.75		
**Anemia**				
No	Ref	–	Ref	–
Yes	2.24 (1.45–3.43)	<0.01	1.67 (1.08–2.58)	**0.02**
AST (U/L)	1.01 (1.00–1.02)	0.11		
TC (mmol/L)	0.89 (0.77–1.04)	0.15		
**Homocysteine**				
<15.3 umol/L	Ref	–	Ref	–
≥15.3 umol/L	2.67 (1.69–4.25)	<0.01	1.83 (1.12–2.99)	**0.02**
**CRP**				
<1.8 mg/dL	Ref	–		
≥1.8 mg/dL	0.91 (0.50–1.67)	0.76		
**HbA1c**				
<6.0%	Ref	–		
≥6.0%	1.05 (0.69–1.58)	0.83		
**Albumin**				
<40 g/L	Ref	–		
≥40 g/L	0.87 (0.57–1.31)	0.49		
**ALT**				
<35 U/L	Ref	–		
≥35 U/L	0.99 (0.53–1.85)	0.98		
**BUN**				
<8.6 mmol/L	Ref	–		
≥8.6 mmol/L	1.50 (0.91–2.46)	0.11		
**Phosphorous**				
<1.5 mmol/L	Ref	–		
≥1.5 mmol/L	1.08 (0.73–1.60)	0.70		
**Sodium**				
<140 mmol/L	Ref	–		
≥140 mmol/L	1.48 (1.00–2.19)	0.05		
**Potassium**				
<3.5 mmol/L	Ref	–	Ref	–
3.5–5.0 mmol/L	0.51 (0.25–1.04)	0.07	0.57 (0.28–1.14)	0.11
≥5.0 mmol/L	0.73 (0.32–1.66)	0.45	0.40 (0.18–0.90)	**0.03**
**TG**				
<1.3 mmol/L	Ref	–		
≥1.3 mmol/L	1.05 (0.70–1.57)	0.82		
**CKD. Stage**				
eGFR < 60 mL/min/1.73 m^2^	Ref	–	Ref	–
eGFR ≥ 60 mL/min/1.73 m^2^	1.35 (0.89–2.06)	0.16	0.61 (0.39–0.94)	**0.03**
**UACR**				
≤30 mg/g	Ref	–		
31–300 mg/g	1.13 (0.74–1.72)	0.57		
>300 mg/g	1.77 (0.95–3.31)	0.07		

*eGFR, estimated glomerular filtration rate; BMI, body mass index; CRP, C-reactive protein; HbA1c, glycosylated hemoglobin; ALT, alanine aminotransferase; AST, aspartate aminotransferase; BUN, blood urea nitrogen; TG, serum total triglycerides; TC, serum total cholesterol; CVD, cardiovascular disease. Bold values indicate the results were statistical significance.*

### Development and Assessment of Predictive Nomogram

#### Internal Validation

**FIGURE 1:**
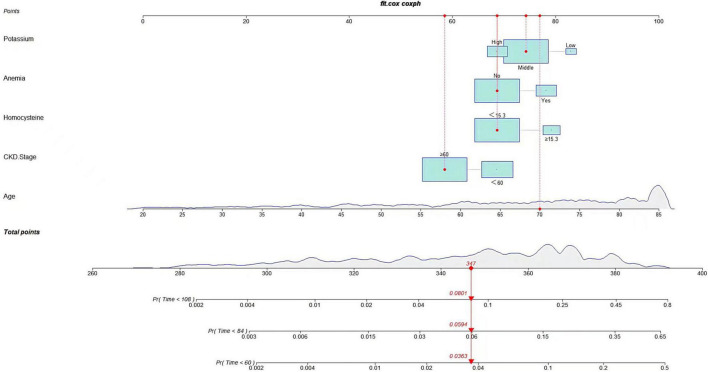
Nomogram to predict the probability of cardiovascular death in CKD patients.

**FIGURE 2:**
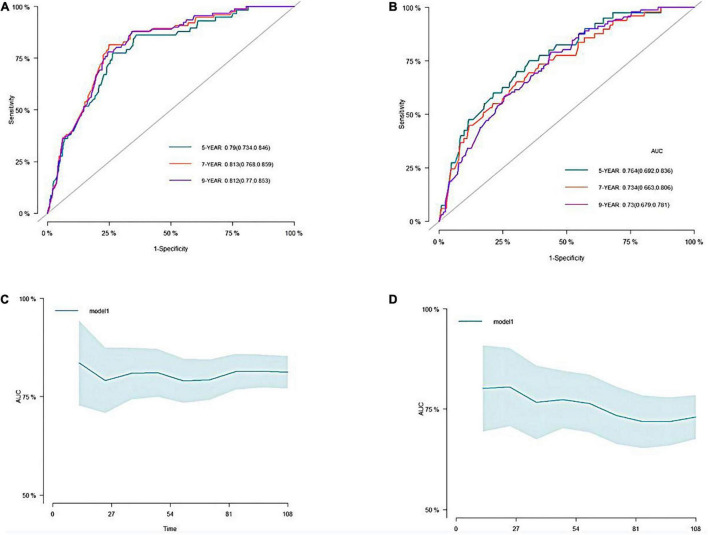
The receiver operating characteristic (ROC) and the area under the curve (AUC) of the nomogram. **(A)** ROC curve of nomogram in the training cohort. **(B)** ROC curve of nomogram in the validation cohort. **(C)** The AUC of nomogram in the training cohort. **(D)** The AUC of nomogram in the validation cohort.

**FIGURE 3:**
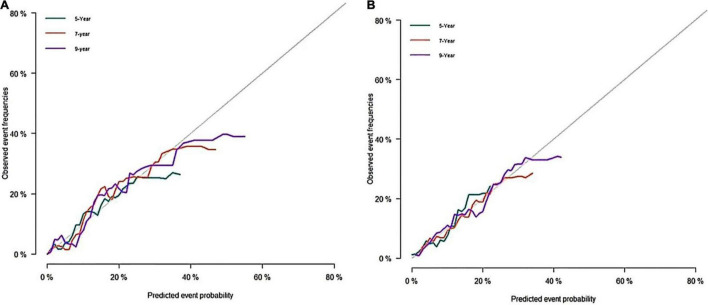
Calibration curves of the nomogram. **(A)** Calibration curve of nomogram in the training cohort; **(B)** calibration curve of nomogram in the validation cohort.

**FIGURE 4:**
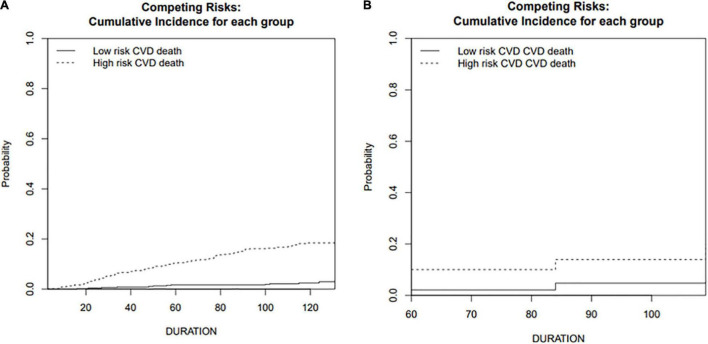
Cumulative incidence function curve of cardiovascular death in training and validation cohorts. **(A)** Training cohort; **(B)** validation cohort.

**FIGURE 5:**
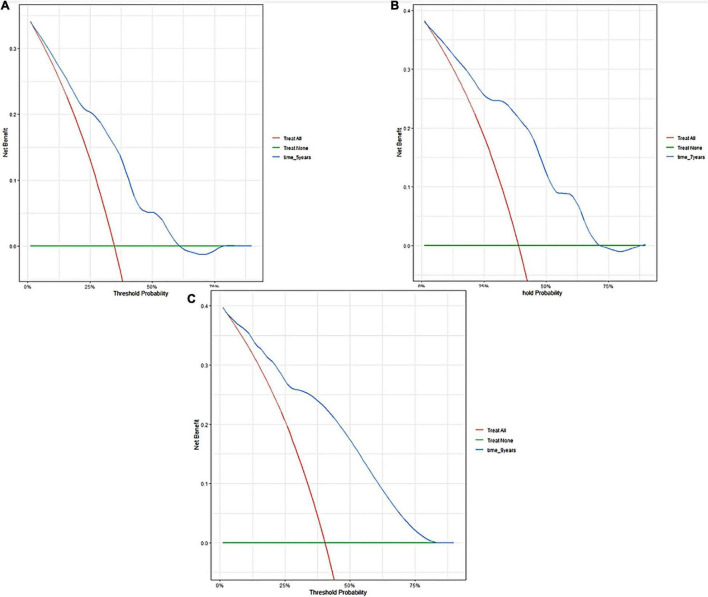
Decision curve analyses of the nomogram in training cohort. Decision curve of the nomogram to predict 5-year **(A)**, 7-year **(B)**, and 9-year **(C)** in training cohort.

A predictive nomogram containing age, homocysteine, CKD stage, potassium levels, and anemia has been established after univariate and backward-stepwise regression (Figure 1). All variables were statistically significant. AUC values of 5–, 7– and 9-years were 0.79 (95% CI: 0.73–0.85), 0.81 (95% CI: 0.77–0.86), and 0.81 (95% CI: 0.77–0.87), respectively (Figure 2). The calibration curves after 1,000 times of bootstraps revealed a good agreement between actual and predicted values (Figure 3). To better reflect the established model’s discrimination. We calculated the total points of each patient according to the established nomogram and divided patients into high- and low-risk CVD death groups. CIF (Figure 4) showed that this nomogram has good discrimination, with a remarkable difference between the two groups (Gray’test: *P* < 0.05). DCA curves indicated that the model provided a net benefit across approximately 60, 70, and 75% of the risk threshold range in 5–, 7–, and 9-years, respectively (Figure 5).

#### External Validation

**FIGURE 6:**
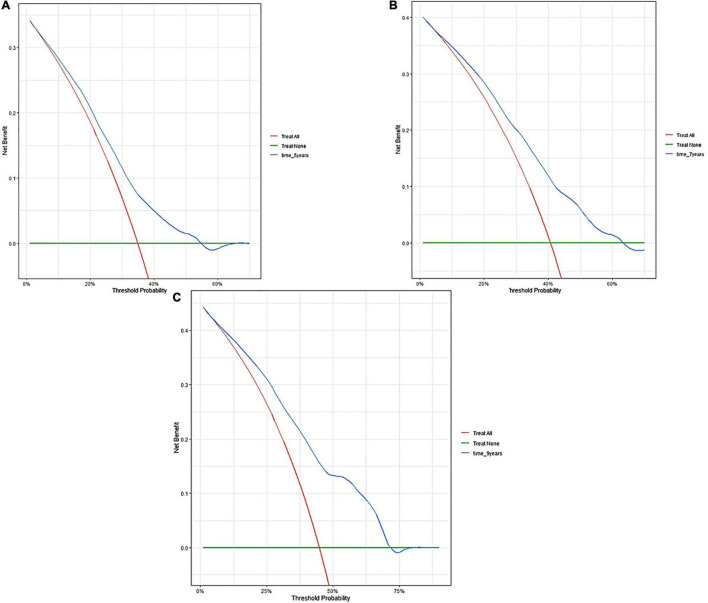
Decision curve analyses of the nomogram in validation cohort. Decision curve of the nomogram to predict 5-year **(A)**, 7-year **(B)**, and 9-year **(C)** in validation cohort.

The external validation also indicated that this nomogram has a good predictive performance. The calibration curve demonstrated that the predicted value basically coincides with the actual value (Figure 3). AUC values of 5, 7 and 9-year OS were 0.76 (95% CI: 0.69–0.84), 0.73 (95% CI: 0.66–0.81), and 0.73 (95% CI: 0.68–0.78), respectively (Figure 2). CIF showed good discrimination between high- and low-risk CVD death groups (Figure 4). DCA curves showed a net benefit across approximately 50, 60, and 70% of the risk threshold range in 5–, 7–, and 9-years, respectively (Figure 6).

## Discussion

Cardiovascular disease is recognized as one of the most prevalent complications of CKD. CVD is also a leading cause of death in CKD patients. The need for optimal risk prediction for CVD prevention is clinically significant. It can distinguish patients based on their different baseline features, identify patients with poor prognosis, and employ medical resources in an efficient manner. This study constructed a novel nomogram based on a competing risk model that included age, homocysteine, CKD stage, anemia, and potassium levels to predict long-term cardiovascular death in CKD patients. To our knowledge, this is the first nomogram to predict cardiovascular death risk in CKD patients. Internal and external validations of the nomogram revealed good predictive abilities, implying that the established nomogram exhibits good external applicability. In this work, we replaced the traditional Cox regression with a competing risk model. Compared with the traditional survival analysis, the competitive event model ensures that the chosen influencing factors are most directly associated with cardiovascular death prognosis (16). CIF demonstrated that our established model could effectively distinguish patients at high risk of CVD death from those at low risk. This is advantageous for identifying patients at high risk of CVD death and then providing appropriate intervention to improve their cardiovascular outcomes.

According to our descriptive statistics for the study population, CVD death accounted for one-third of all deaths, ranking as the first most common cause of death for CKD patients. Previous studies have revealed that mild-to-moderate CKD patients (stages 3A and 3B) have a considerably higher risk of cardiovascular mortality than patients with kidney failure (17). These findings indicate that core issues that clinicians should prioritize the risk of cardiovascular diseases over the risk of reaching kidney failure requiring renal replacement therapy. As for the key factors to consider after selection, our study revealed that age is the main factor causing CVD death in CKD patients since it accounted for most points in established nomograms. According to the 2013 global burden disease (18), the risk of cardiovascular death due to population aging has increased by 40.8% over the last two decades. Among them, death caused by arrhythmia and hypertension mostly increased. The assessment of CVD risk in the elderly is also important. The 2021 ESC guidelines (19) advocated routine cardiovascular risk assessment in men over 40 and women over 50. Our study revealed that, besides age, potassium levels also play a major role in determining cardiovascular outcomes. Interestingly, hypokalemia appears to be more likely to contribute to cardiovascular mortality. This could be due to the lower number of patients with higher blood potassium levels in the enrolled population, whereas patients with mild elevations in blood potassium levels have a correspondingly high potassium tolerance. SCREAM trial (20) enrolled 78,997 patients with stages 3–5 CKD and indicated that patients with poor kidney function seem to have better potassium tolerance. This may explain why our study found that hyperkalemia did not significantly increase the risk of cardiovascular outcomes. Anemia is considered as one of the predictors of cardiovascular events in CKD patients. Some scholars have proposed the concept of “cardio-renal-anemia syndrome” (21, 22), which aims to emphasize the critical function of anemia in the cardiorenal axis. A large cohort study in the United States discovered that the risk of developing various cardiovascular diseases increased when hemoglobin levels decreased in CKD patients (23). An epidemiological study found that after 3 years of observation of patients with stage 3 CKD, those with anemia had a twice greater incidence of cardiovascular events than those who were not anemic (24). CKD stage is known to be a direct indicator of cardiovascular events. In our study, cardiovascular death risk increases significantly from stage 3 CKD. This finding is consistent with previous studies, demonstrating that cardiovascular death risk was approximately twice as high in patients with stage 3 CKD (eGFR 30–59 mL/min per 1.73 m^2^) and three times higher in stage 4 (15–29 mL/min per 1.73 m^2^) than in individuals with normal kidney function (25, 26). Homocysteine is generally considered to be linked to cardiovascular and cerebrovascular prognosis. According to a meta-analysis, elevated homocysteine levels are an independent predictor of cardiovascular mortality (27). Together with our findings, we recommended that homocysteine should be routinely measured in CKD patients to detect their prognosis of cardiovascular outcomes.

It is undeniable that our prediction model has some limitations. First, all data of our enrolled patients were obtained from NHANES database, although we validated using data from many time points. Additionally, multicenter clinical validation is required to assess the nomogram’s external applicability. Second, due to limited data of NHANES database, some crucial indicators of cardiovascular prognoses, such as brain natriuretic peptide or cardiac troponin, were not investigated. Third, our study included a substantial number of non-dialysis patients. The prediction efficacy of the developed nomogram for dialysis patients should be further validated.

## Conclusion

Through a competing risk model, we established a novel nomogram incorporating age, homocysteine, CKD stage, anemia, and potassium levels. The internal and external validations revealed the good predictive performance of this nomogram. Additional data on CKD patients should be used in the future to confirm the nomogram’s prediction ability.

## Data Availability Statement

The raw data supporting the conclusions of this article will be made available by the authors, without undue reservation.

## Ethics Statement

Ethical review and approval was not required for the study on human participants in accordance with the local legislation and institutional requirements. Written informed consent for participation was not required for this study in accordance with the national legislation and the institutional requirements. Written informed consent was not obtained from the individual(s) for the publication of any potentially identifiable images or data included in this article.

## Author Contributions

NL and LZ contributed to the concept and design of this study. NL, YX, JZ, GZ, and YZ were responsible for statistical analysis and writing of the report. WH assisted in statistical analysis. MY, LX, EZ, and WS reviewed the article and provided critical feedback to improve and structure the report. All authors read and approved the final manuscript.

## Conflict of Interest

The authors declare that the research was conducted in the absence of any commercial or financial relationships that could be construed as a potential conflict of interest.

## Publisher’s Note

All claims expressed in this article are solely those of the authors and do not necessarily represent those of their affiliated organizations, or those of the publisher, the editors and the reviewers. Any product that may be evaluated in this article, or claim that may be made by its manufacturer, is not guaranteed or endorsed by the publisher.

## Footnotes

1
https://www.rproject.org/
